# Classification method based on surf and sift features for alzheimer diagnosis using diffusion tensor magnetic resonance imaging

**DOI:** 10.1038/s41598-025-92759-2

**Published:** 2025-03-21

**Authors:** Nourhan Zayed, Ghaidaa Eldeep, Inas A. Yassine

**Affiliations:** 1 Computer and Systems Department, Electronics Research Institute, Cairo, Egypt; 2 Mechatronics Engineering, The British University in Egypt, Cairo, Egypt; 3 Systems and Biomedical Engineering, Cairo University, Cairo, Egypt

**Keywords:** Alzheimer ’s disease (AD), SIFT features and SURF features, Diffusion tensor imaging (DTI), Bag of words, Hippocampus, Amygdala, Neurological disorders, Medical imaging, Engineering

## Abstract

Alzheimer’s disease (AD), the most common dementia in the elderly, poses a challenge for early diagnosis due to its progressive nature and hidden microstructural changes. While traditional T_1_ and T_2_ weighted MRI can assess macro-structural brain atrophy, diffusion tensor imaging (DTI) unveils these hidden microstructural alterations. This study explores the use of DTI data, specifically visual patterns in Fractional Anisotropy (FA), Mean Diffusivity (MD), and Radial Diffusivity (RD) maps, to characterize AD progression. This paper proposes a computer-aided diagnosis (CAD) framework employing SIFT and SURF descriptors and a bag-of-words approach to build AD-specific signatures for the hippocampus region, known to be heavily affected by the disease. These signatures are extracted from MD, FA, and RD maps and used to differentiate between AD, mild cognitive impairment (MCI), and normal controls (NC) in both multiclass and binary classification scenarios. Additionally, we investigate late fusion of visual map features for enhanced decision-making. The experiments were accomplished with a subset of participants from the Alzheimer’s Disease Neuroimaging Initiative (ADNI) dataset formed of AD patients (*n* = 35), Early Mild Cognitive Impairment (EMCI) (*n* = 6), Late Mild Cognitive Impairment (LMCI) (*n* = 24) and cognitively healthy elderly Normal Controls (NC) (*n* = 31). Promising preliminary results demonstrate the potential of the proposed system as a useful tool to capture the AD leanness with achieving accuracies of 87.5%, 87.4%, 89%, and 95.2% for MD, FA, RD, and fusion of features respectively for the multiclass system using SIFT features. Using FA features for binary discrimination achieves 97.5%. Moreover, the fusion based on the decision level model reached an accuracy of 93.3% AD/MCI, 95.7% AD/NC, and 93.3% MCI/NC (96.2 ± 3.6 MCI vs. NC, 97.5 ± 5 AD vs. NC). Furthermore, fusion of features led to a noteworthy precision boost of 96%. These findings suggest that our DTI-based CAD framework holds promise as a reliable and accurate tool for capturing AD progression, paving the way for earlier diagnosis and potentially improved patient outcomes.

**Supplementary Information:**

The online version contains supplementary material available at 10.1038/s41598-025-92759-2.

## Introduction

Alzheimer’s disease (AD), a chronic and progressive neurodegenerative disease, poses a major threat with its staggering prevalence. In 2015, it ranked as the sixth leading cause of death in the US, affecting nearly 44 million people worldwide^1^. Direfully, experts predict this number to double within the next two decades, with one in 85 individuals estimated to suffer from AD by 2050^1–3^. Characterised by memory loss and behavioural changes due to brain cell death, AD’s first symptoms can appear as early as the 30–60 s^4,5^. The initial manifestation varies among individuals, but memory problems often serve as a telltale sign. As the disease progresses, patients may enter the stage of Mild Cognitive Impairment (MCI), experiencing significant memory loss and other cognitive difficulties while still managing daily activities with minimal support^5^. This stage can last for 20 to 30 years, making it the longest phase of AD^2^. The disease typically progresses through various stages: preclinical, mild (early-stage), moderate, and severe (late-stage). This final stage, lasting around five years, often leads to death.

With the alarmingly rising AD prevalence, early diagnosis and treatment become crucial battlegrounds in modern medicine. Advances in neuroimaging and biomarker identification are revolutionising our understanding of the disease. Structured magnetic resonance imaging (MRI), with its high resolution, plays a key role in visualising brain anatomy and measuring neurodegeneration through new biomarker-based definitions^6,7^. Early research in this domain has focused on analysing MRI images to assess brain atrophy, particularly in regions like the hippocampus and amygdala^8–12^. To do this, you have to measure changes in volume or look at changes in the MRI signal caused by changes in the tissue^13^, like white matter hyperintensities. Recent studies have confirmed elevated white matter hyperintensity loads in AD patients, especially in specific brain regions^11,12^.

Regional volume analysis has also demonstrated significant atrophy in the hippocampus and surrounding structures of AD patients compared to healthy individuals^8–10,14,15^. The growing global population ageing will likely see 80% of individuals experiencing dementia, with AD taking centre stage^1^. While traditional MRI excels at detecting large-scale brain shrinkage, a crucial biomarker, it remains blind to the intricate microstructure and misses crucial subtle changes in brain tissue fibres, axons, and cell boundaries. Diffusion Tensor Imaging (DTI) is becoming a powerful tool to capture these hidden alterations. This could potentially unlock a new frontier in early AD diagnosis and paving the way for more effective interventions.

DTI measures the movement of water molecules within brain tissue, along the white matter fibres. In healthy brains, water movement is restricted by intact cell membranes and organised fibre tracts, leading to directional diffusion (anisotropy). But in AD-affected regions, cellular damage and fibre disorganization break down these barriers, allowing water to diffuse more freely in all directions (reduced anisotropy). By analysing DTI-derived anisotropy maps, researchers can pinpoint areas of neurodegeneration. Traditionally, there are different types of diffusion tensor imaging (DTI) maps/measures like Mean Diffusivity (MD). Fractional Anisotropy (FA), and Radial Diffusivity (RD)^15,16^.

The Hippocampus and Amygdala are considered the utmost smitten part in terms of shape by Alzheimer’s retrograding^8,17–23^. As The hippocampus, crucial for memory and navigation^19–21,24^. Whereas the Amygdala plays a pivotal function in the emotional processing such as: memory associated with emotions, and emotional stimuli adaptive responses^23^. Their early involvement in AD makes them a sensitive marker for disease progression. Studies have shown a strong correlation between hippocampal shape alterations and memory decline, further solidifying its role as a diagnostic tool. Thus, finding new biomarkers is changing our understanding of AD, especially zooming out on these microstructures’ changes, within these regions (associated with cognitive impairment of patients). Otherwise, the existing challenge for modern neuroimaging is to help diagnose early AD and MCI patients. This will reflect the disease stage and the predictive progression of mild cognitive.

Recent research focuses on extracting features from T1-weighted MRI images using Gaussian map descriptors, with 74.6% accuracy in distinguishing between healthy individuals and those with any form of cognitive impairment and 98.9% accuracy in differentiating between AD patients and individuals with MCI^11,12^. Other studies highlight the synergy between effective feature extraction and sophisticated algorithms in unlocking the mysteries of AD^8,13,14,25,26^. Although these research results are promising from the prospective of macrostructural level, the micro-structural changes remained invisible in the anatomical scan and remains less developed^2^. The latter can be delineated by other MRI modalities such as Diffusion Tensor Imaging (DTI).

On this basis, researchers have explored diverse approaches to achieve better results using the visual appearance of different DTI maps. Dyrba et al.^27^ leveraged machine learning on a large dataset, analysing both tissue density and water diffusion properties (MD and FA) to differentiate AD from healthy individuals with an accuracy of 80–83%. Chen et al.^28^ introduced a novel technique that extracts unique local features from brain scans, achieving accuracy between 70% and 87% in identifying AD, Parkinson’s, and bipolar disorder. Ahmed et al.^29^ employed visual maps of water diffusion to create an “AD signature” with 86% accuracy.

Meanwhile, Schouten et al.^30^ cast a wider net, analysing not just brain anatomy but also its activity and connections. By combining various measures, they achieved an impressive 95% accuracy in distinguishing AD from healthy brains. Ahmed et al.^31,32^ further explored the power of combining information. They extracted features from both brain anatomy (MRI) and water diffusion (DTI), particularly focusing on the hippocampus, a region heavily affected by AD. This multimodal approach yielded accuracy exceeding 90% in differentiating AD from healthy and Mild Cognitive Impairment (MCI) individuals.

Deng & Wang research^33^ tackles the rising concerns surrounding Alzheimer’s disease (AD) by proposing a novel machine learning model for its early and accurate diagnosis. Combining the power of diffusion tensor imaging (DTI) and T1-weighted images, the model employs wavelet and texture-based approaches to extract key features from MRI scans, like fingerprints revealing the disease’s telltale signatures. These extracted features are then fed into powerful machine learning algorithms like Support Vector Machines (SVM) and Linear Discriminant Analysis (LDA), allowing them to classify and diagnose AD with remarkable accuracy^33^. Several studies^27^ have explored the use of machine learning to analyze brain scans and differentiate between AD, MCI, and healthy individuals. Dyrba et al., for example, achieved an accuracy of 80–83% in classifying subjects based on brain diffusion properties^27^. Other studies^27^, like Ben et al., focused on visual patterns in diffusion maps, reaching an accuracy of 86.73% for AD vs. healthy cases and 77% for MCI vs. healthy cases. Notably, Ben et al.‘s multimodal approach (combining different types of brain scan data) further improved accuracy to 90.2% for AD vs. healthy cases^32^. Literature suggests, nevertheless, that no single biomarker provides sufficient information to capture the inert symmetry of disease contrary to the realized spectrum. Traditionally, researchers have relied on DTI measures like MD and FA^33^. These measures capture the movement of water molecules within brain tissue, providing insights into the integrity of white matter fibre tracts, a key target of AD. Additionally, researchers have extracted various local features from these measures, such as texture descriptors or shape analysis, further refining the search for disease-specific patterns. While MD and FA have been workhorses in AD detection, another DTI measure, RD, hasn’t received as much attention. RD reflects water diffusion perpendicular to the fibre tracts, potentially offering unique insights into AD’s effects on tissue microstructure. Exploring the potential of RD maps, alongside established measures and local features, could unlock new avenues for early and accurate AD diagnosis.

Thus, this paper proposes a novel system to build meaningful AD-medical signatures for computer-aided diagnosis (CAD) framework. Leveraging the visual features of DTI’s Mean Diffusivity (MD), Fractional Anisotropy (FA), and Radial Diffusivity (RD) maps (i.e. visual appearance is related to the voxel intensity value in the image), calculated for the hippocampus and amygdala regions, for AD patient discrimination based on binary and multiclass systems using SIFT and SURF descriptors to build AD-signature using Bag of-words a (BOW) approach. Scale-Invariant Feature Transform (SIFT) and Speeded Up Robust Features (SURF), are utilized in this work. Both descriptors excel at identifying and characterising local image features within the selected ROI. While SIFT offers robust scale and rotation invariance, SURF boasts faster computational efficiency.

SIFT (Scale-Invariant Feature Transform) and SURF (Speeded-Up Robust Features) are important in the classification of Alzheimer’s disease using medical images because they provide robust and invariant features that can be used to analyze and differentiate between healthy and pathological brain structures. Here’s why they are significant: (1) **Invariance to Scale and Rotation: SIFT** and **SURF** are designed to detect and describe local features in images that are invariant to scale and rotation. This is particularly important in medical imaging, where the size and orientation of brain structures can vary across patients or even within the same patient over time. For Alzheimer’s diagnosis, this invariance ensures that features extracted from brain scans (e.g., MRI, DTI, or CT) remain consistent regardless of the imaging conditions or patient positioning. (2) **Robustness to Noise**: Medical images often contain noise due to imaging artifacts, low resolution, or patient movement. SIFT and SURF are robust to noise, making them suitable for extracting reliable features from noisy medical images. This robustness is critical for accurately identifying subtle changes in brain structures associated with Alzheimer’s disease, such as hippocampal atrophy or cortical thinning. (3) **Local Feature Extraction**: SIFT and SURF focus on local features, which are crucial for identifying specific regions of interest (ROIs) in the brain that are affected by Alzheimer’s disease. For example, they can detect changes in the hippocampus, amygdala, entorhinal cortex, or ventricles, which are key biomarkers for the disease. By extracting local features, these algorithms can capture fine-grained details that might be missed by global feature extraction methods. (4) **Ability to Handle Complex Patterns**: Alzheimer’s disease involves complex patterns of brain atrophy and structural changes. SIFT and SURF can detect and describe these patterns by identifying keypoints and their descriptors, which can then be used for classification. These features can be fed into machine learning models (e.g., SVM, random forests, or neural networks) to classify images as healthy, mild cognitive impairment (MCI), or Alzheimer’s disease. (5) **Complementary to Deep Learning**: While deep learning methods (e.g., CNNs) have become dominant in medical image analysis, SIFT and SURF can still play a complementary role. They can be used to preprocess images, extract handcrafted features, or provide additional input to deep learning models, improving overall classification performance. (6) **Interpretability**: Unlike deep learning models, which are often considered “black boxes,” SIFT and SURF features are interpretable. Researchers and clinicians can visualize the keypoints and descriptors to understand which regions of the brain are contributing to the classification, aiding in diagnosis and research. There are many applications in Alzheimer’s Diagnosis: **Hippocampal Atrophy Detection**: SIFT and SURF can identify changes in the hippocampus, a region heavily affected by Alzheimer’s^31,32^. **Ventricular Enlargement**: These features can detect changes in ventricle size, which is a common biomarker for the disease^31,33^. **Cortical Thinning**: They can help identify thinning of the cerebral cortex, another hallmark of Alzheimer’s progression^31–33^. To our knowledge, none of the researchers before used RD nor FA maps as visual intensities values and a fusion of three maps together as features level or decision level. The dataset used, in this study, is a subset of Alzheimer’s disease Neuroimaging Initiative (ADNI) database, a complete description of ADNI is available at http://adni.loni.usc.edu/. The rest of the paper is organised as follows: the materials and methods are discussed in Sect. 2. The experiments and results are reported in Sect. 3. Finally, Sect. 4 concludes the work and outlines its perspectives.

## Materials and methods

**Fig. 1:**
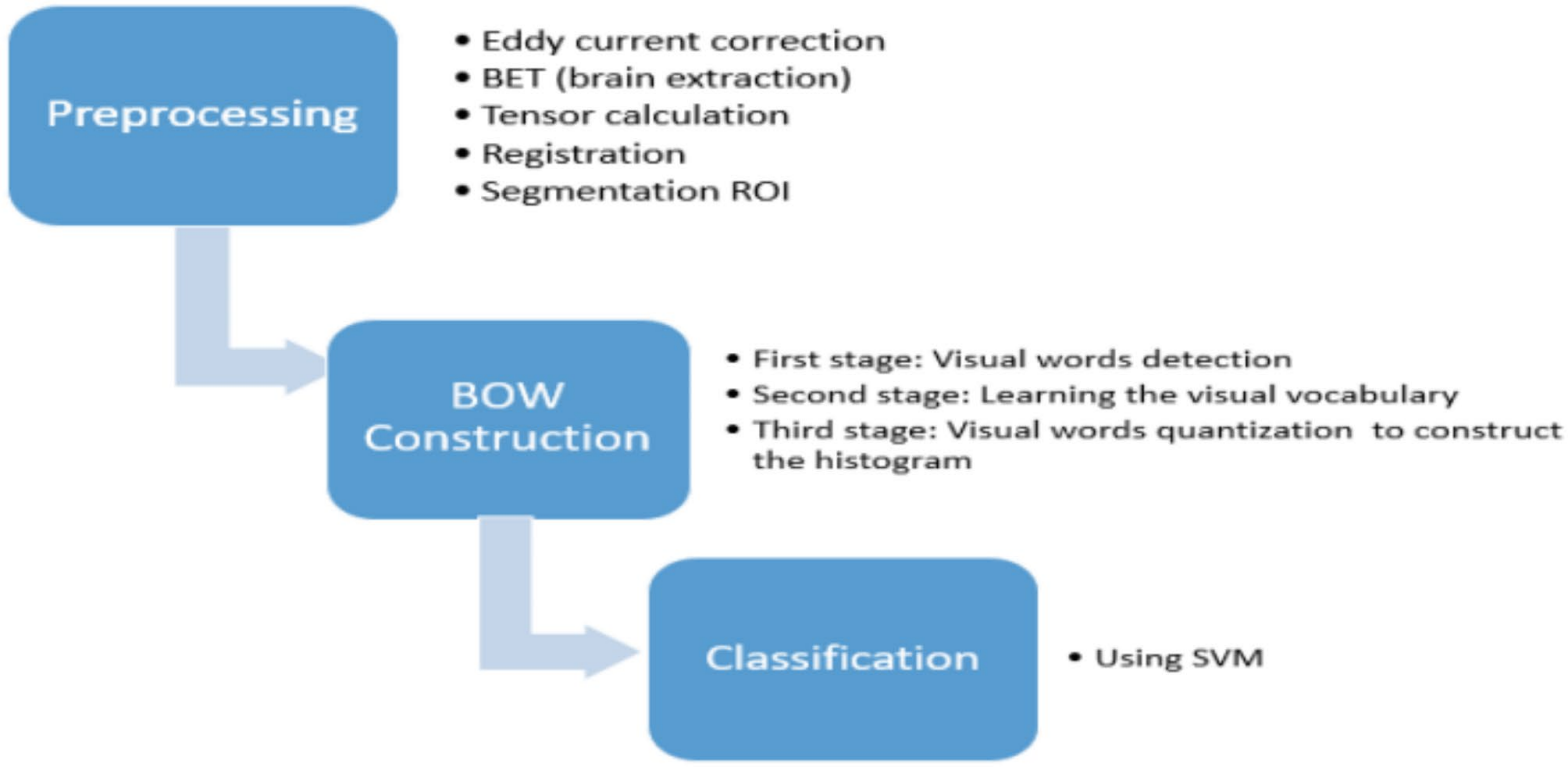
Proposed system for AD Classification using SURF and SIFT features calculated from the visual MD, FA, and RD diffusion tensor imaging maps.

This section will present the proposed system workflow for our framework (see Fig. 1). Firstly, exploring the dataset used in this study and its description, data preprocessing and consequently the other algorithm steps. This includes an overview of how to extract the used ROI, selecting the features, and a brief about the BoW approach. Followed by the performance metrics used to evaluate the proposed classification systems are given. This study seeks to construct a visual signature of the hippocampus, revealing its potential decline in Alzheimer’s disease from the MD, FA and RD images. To achieve this, this paper will meticulously prepare the brain scans by filtering out noise, aligning them to a standard template, and finally isolating the hippocampus itself. This thorough preprocessing lays the groundwork for analysing the hippocampus’s unique signature and its potential connection to the disease.

### Dataset description

The DTI datasets used in this study was obtained from Alzheimer’s disease Neuroimaging Initiative (ADNI) initiative. In this study. All participants were scanned on the same scanner model, 2) Standardized image acquisition protocols were strictly adhered to. Each ADNI subject is formed of 5 T2-weighted images with no diffusion sensitization (b = 0 images) and 41 diffusion-weighted images (b = 1000 s/mm^2^), acquired for each DTI slice. Table 1 shows all the scanning parameters. A complete description of ADNI is available at http://adni.loni.usc.edu/. Moreover, the data access requests are to be sent to http://adni.loni.usc.edu/data-samples/access-data/. The Mini Mental State Examination (MMSE), mentioned in Table 2, is a psychological test quantifying the cognitive function but isn’t enough to make a full and accurate Alzheimer diagnosis because most questions test only memory and recall. Our study includes ninety-six subjects (96), Table 2 shows all the demographic descriptions about the dataset used. and the results of running t-test statistical data analysis. The general typical selection criteria for patients in ADNI datasets is designed to ensure the inclusion of subjects who are representative of the population being studied (e.g., healthy normal controls, individuals with mild cognitive impairment (MCI), and those with Alzheimer’s disease (AD)). The inclusion criteria include: (1) **Age**: Typically, participants are aged 55–90 years. (2) Cognitive Status: Participants must meet specific cognitive and clinical criteria for their respective diagnostic group (HC, MCI, or AD). (3) **Imaging Availability**: Participants must have completed the required imaging protocols (e.g., T2-weighted and diffusion-weighted imaging (DTI) as described in the study). (4) **Informed Consent**: All participants must provide written informed consent to participate in the study. while exclusion criteria include: (1) **Major Neurological or Psychiatric Disorders**: Participants with conditions other than Alzheimer’s disease that could affect cognitive function (e.g., stroke, Parkinson’s disease, major depression). (2) **Significant Medical Conditions**: Conditions that could interfere with the study, such as severe cardiovascular disease or uncontrolled diabetes. (3) **Contraindications for MRI**: Participants with metal implants, claustrophobia, or other contraindications for MRI scanning are excluded. (4) **Medications**: Use of certain medications that could affect cognitive function or imaging results may lead to exclusion.

**Table 1:** DTI scan parameters.

Dataset	ADNI
Scanner	3-Tesla GE Medical Systems scanners
Scan matrix	256 × 256 × 59 voxels per volume
Voxel size	1.3 × 1.3 × 2.7 mm^3^
TR (ms)	9050 ms
TE	61.8 ms
Diffusion parameters	41 diffusion-weighted images (b = 1000 s/mm^2^)

**Table 2:** Demographic description of the ADNI group. Values are denoted as mean ± standard deviation (STD).

Type	AD	NC	MCI	*p*-value for group difference		
				NC vs. MCI	NC vs. AD	MCI vs. AD
Number	35	31	30	**–**	**–**	**–**
Age	73 ± 7	69 ± 5	76 ± 4	**0.0001**	**0.0104**	**0.0422**
Gender (M/F)	20/15	20/11	19/11	**–**	**–**	**–**
MMSE	23.3 ± 0.9	29 ± 1.2	26.5 ± 0.7	**0.0001**	**0.0001**	**0.0001**

Bold entries signify P B 0.05.

### Data preparation and preprocessing

This section details the essential steps employed in preparing diffusion tensor imaging (DTI) data for subsequent segmentation of the hippocampus, key region implicated in memory and cognition (as shown in Fig. 1). These steps are performed using FSL, a powerful toolkit developed at Oxford University specifically for analysing brain scans^34^. Each step plays a vital role in ensuring accurate analysis later. Brain Extraction Tool (BET) inside the FSL package is used for skull stripping, while DTI fit module is used for the diffusion tensor estimation^35,36^. The steps are as follows:

Firstly, Noise Attenuation: DTI data can be corrupted by various sources of noise, including head motion, thermal fluctuations, and scanner artifacts. To mitigate these effects, eddy current correction and temporal filtering techniques are applied. FSL’s FDT toolset is utilized for this purpose, ensuring improved signal-to-noise ratio and enhanced image clarity.

Secondly, Skull Stripping: Non-brain tissue, primarily the skull, can pose a substantial obstacle to accurate ROI (region of interest) segmentation. Employing FSL’s Brain Extraction Tool (BET), we meticulously remove the skull while preserving crucial near-skull white matter structures. This step ensures accurate delineation of brain regions and minimizes potential segmentation errors.

Thirdly, Diffusion Tensor Calculation: Following noise reduction and skull stripping, diffusion tensor estimation is performed for each voxel within the brain. This step, utilizing FSL’s DTI fit module, yields quantitative measures of water diffusion characteristics, essential for characterising white matter integrity and analysing potential AD-related alterations.

Fourthly, Spatial Normalization: Inter-individual anatomical variations necessitate alignment of DTI data to a standard reference space. This enables consistent and reliable comparisons of ROI volumes and diffusion metrics across subjects. FSL’s FLIRT toolset accomplishes this alignment through non-linear registration to an MNI152 template, reducing anatomical variability and facilitating robust statistical analysis.

**Fig. 2:**
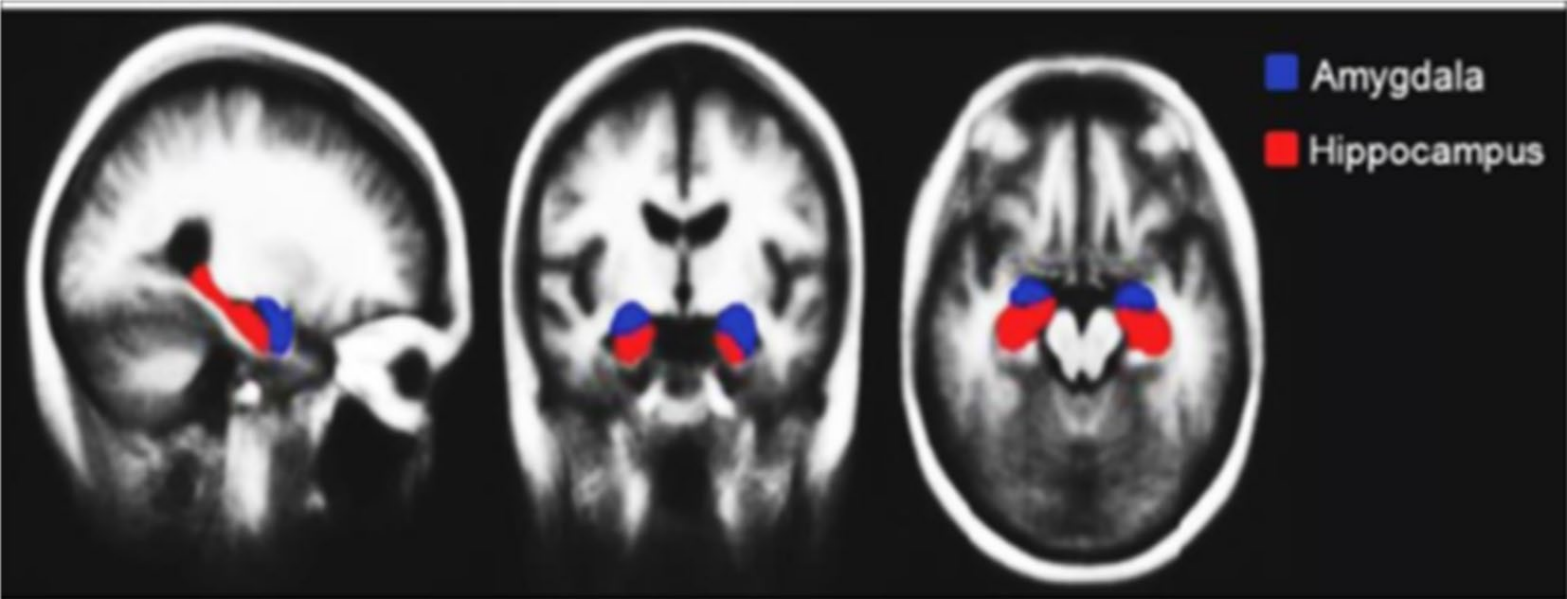
Hippocampus and Amygdala position.

**Fig. 3:**
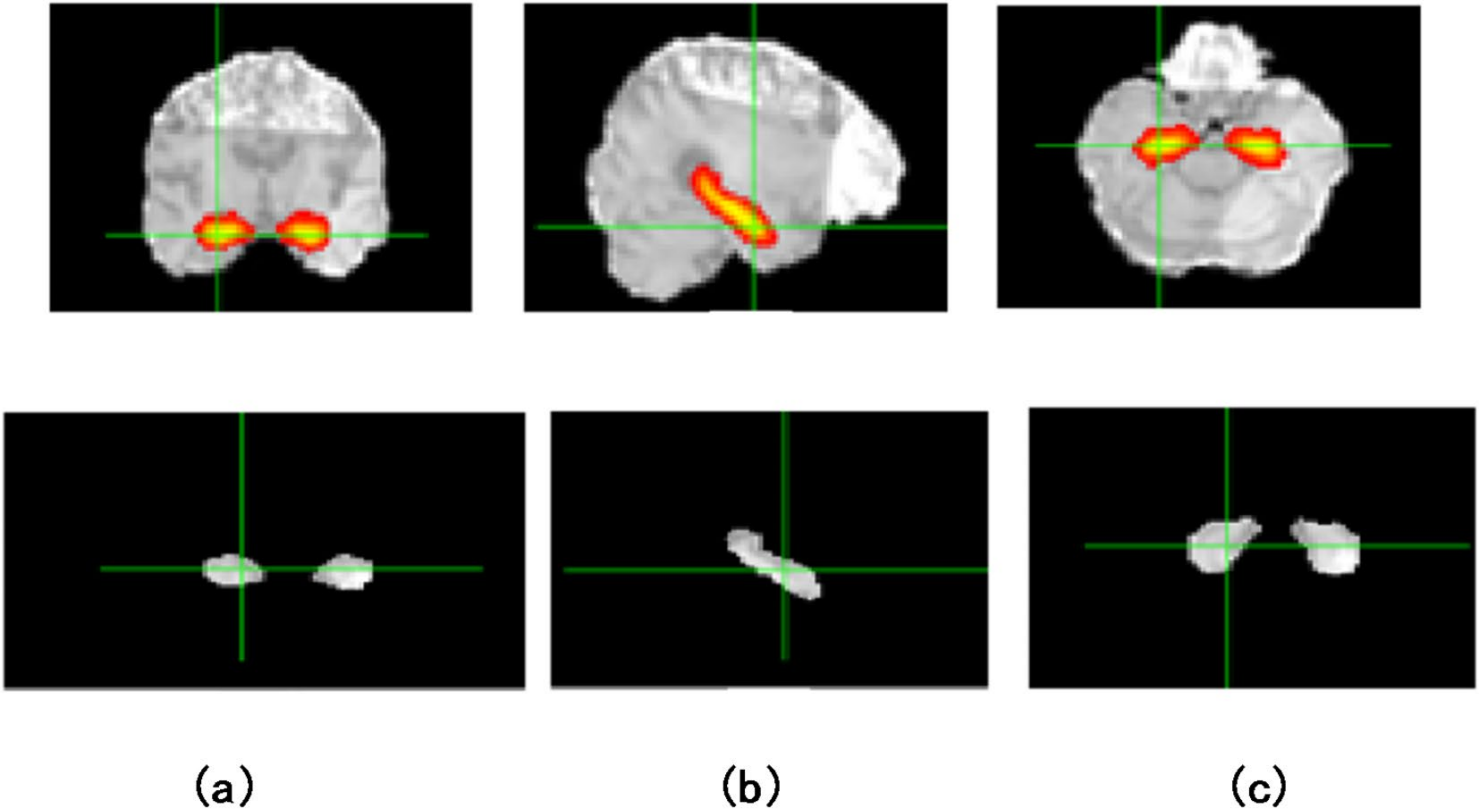
Hippocampus ROI selection in three planes of an MRI slice and Segmented Hippocampus region for (**a**) Axial, (**b**) Sagittal and (**c**) Coronal planes.

Finally, Region of Interest Segmentation: the preprocessed DTI data is utilized for segmentation of the hippocampus, as shown in Figs. 2 and 3. FSL’s FIRST toolset, employing a probabilistic atlas-based approach, enables accurate and reliable extraction of this key region. To form the ROI later to be analyzed and diffusion parameter calculated for further investigating to check the potential involvement in AD pathology.

### Feature extraction and bow approach

Following the meticulous selection of Regions of Interest (ROIs), particularly the hippocampus, the next crucial step in this research involves extracting and analysing distinctive visual (i.e., Values of intensities of these maps) features within these regions. These features act as “fingerprints” potentially revealing the presence or absence of Alzheimer’s disease (AD). This section discusses the employed techniques and rationale behind visual signature construction for the hippocampus.

Firstly, Feature Descriptors: Two powerful feature descriptors, Scale-Invariant Feature Transform (SIFT) and Speeded Up Robust Features (SURF), are utilized in this work. Both descriptors excel at identifying and characterising local image features within the hippocampus ROI. While SIFT offers robust scale and rotation invariance, SURF boasts faster computational efficiency.

Secondly, Building the “Visual Vocabulary”: Bag of Words (BoW) Model^37^. Given the varying number of extracted features per slice and subject, a robust model is needed to represent the overall feature distribution within the hippocampus. The Bag of Words (BoW) model serves as the chosen paradigm. Here, each unique feature (“visual word”) is assigned a “word” in the vocabulary, and its occurrence frequency across the ROI is captured in a “volume-signature histogram”. This histogram essentially becomes a unique fingerprint of the hippocampus, potentially holding clues about AD presence.

**Fig. 4:**
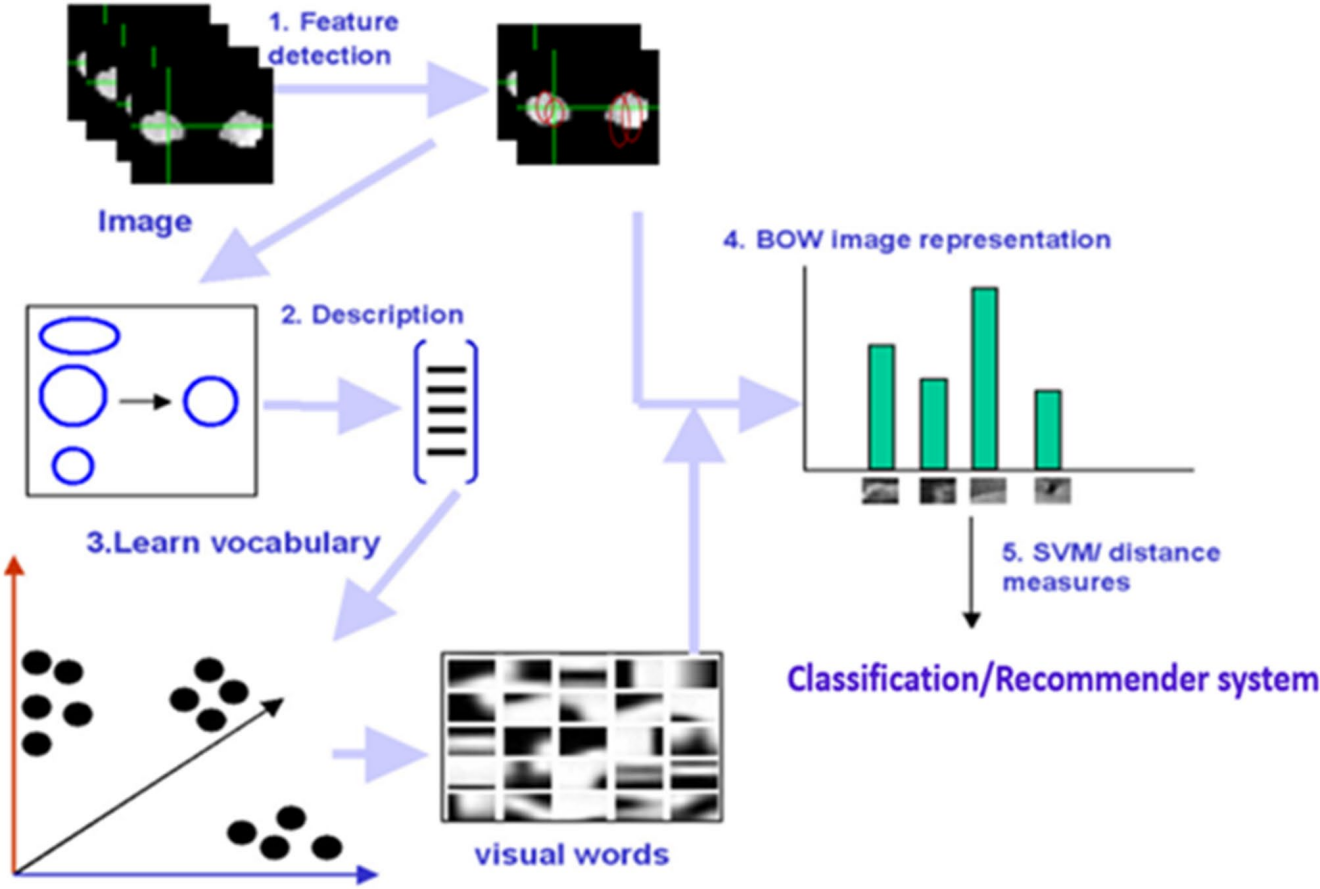
BoW approach.

Thirdly, extracting the Volume-Signature Histograms, a model represents the whole volume or selected ROI (hippocampus) as a histogram of the occurrence of quantized visual features which are called “visual words.”: The key steps involved in this is outlined in Fig. 4 visually. (1) Feature Detection: SIFT and SURF descriptors are computed for each slice of the hippocampus ROI across FA, MD, and RD maps. (2) Based on^38^, the four main stages to compute SIFT descriptors are: Scale-space extrema detection to identify potential “interest points.“, keypoint localization for precise point location, orientation assignment to ensure consistent feature representation, and keypoint descriptor calculation to capture unique local image properties. SURF is similar to SIFT features in properties and steps. It is based on the Hessian matrix (H) to locate interest point^39^. 3)Vocabulary construction: Identified features are clustered into distinct “visual words,” forming the vocabulary for the BoW model. 4) Histogram formation: The occurrence frequency of each visual word within the hippocampus ROI is counted and compiled into the volume-signature histogram.

This systematic approach extracts valuable local features from the hippocampus and builds a comprehensive visual signature using the BoW model. By analysing these signatures across subjects with and without AD, in this paper researchers can potentially uncover subtle differences associated with the disease, paving the way for more understanding of the disease.

### Feature reduction, classification, and performance evaluation

This section dives into two crucial steps employed in this study: dimensionality reduction and supervised learning for classification. These steps are critical for effectively analysing the extracted visual signatures of the hippocampus in the context of Alzheimer’s disease (AD).

#### Dimensionality reduction

The vast number of features extracted from the hippocampal ROIs, coupled with the relatively small data volume, necessitates dimensionality reduction. This step helps mitigate the risk of overfitting. Dimensionality reduction is appointed based on a well-known feature selection approach named Fisher Score^40,41^.

#### Fisher score based feature selection

This study utilizes the well-established Fisher Score approach for feature selection^41^. The Fisher Score effectively measures the discriminatory power of each feature, indicating its usefulness in distinguishing between subjects with and without AD. By selecting features with higher Fisher Scores, we effectively reduce the dimensionality while retaining critical information relevant to AD classification.

#### Supervised learning with SVM-RBF

Support Vector Machine (SVM) is employed as the supervised learning algorithm for classification. SVMs excel at finding a hyperplane that optimally separates distinct data classes in high-dimensional spaces. This study specifically utilizes an SVM with a Radial Basis Function (RBF) kernel, implemented using the LIBSVM package from National Taiwan University^42^. This combination allows for non-linear decision boundaries, potentially capturing complex relationships between the extracted features and AD presence.

#### Performance evaluation with ROC curve

The Receiver Operating Characteristic (ROC) curve evaluates the performance of the proposed classification system. ROC curves effectively assess the diagnostic accuracy of tests by depicting the trade-off between sensitivity and specificity. This metric is particularly valuable in the context of AD research, allowing for clear comparisons between different classification models and providing insights into their ability to correctly distinguish AD patients from healthy individuals.

## Results and discussion

The paper framework is done by conducting two experiments: multiclass classification and binary classification. Bi-wise comparisons are done, in between different dataset categories (NC vs. MCI, NC vs. AD, MCI vs. AD), using SURF and SIFT features, as shown in Table 3, and 4, respectively. The algorithm’s performance will be measured by evaluation based on well-known metrics accuracy, sensitivity, and specificity based on ten folds cross-validation to illustrate the system robustness. We have included the results of the 10-fold cross-validation analysis in Tables 3 and 4 reporting the average performance and standard deviation across the folds.

Also, ROC curves are presented for MD, FA, RD maps and for the fusion between them.

This manuscript recounts fusion in two levels: (1) Feature fusion, (2) Decision level fusion. Feature level fusion is done by concatenating the features extracted from the MD, FA and RD maps together in one feature vector before constructing BoW.

Otherwise, decision level fusion is based on a majority vote.A majority vote means that FA, RD, and MD maps each of them has the decision of classification and comparing their final decisions together to get the final decision, i.e., if the decision according to FA is class one and the decision of MD is class two. So, the decision of RD classification will be used to get the final vote. If RD votes to one, the final classification will be class one but if RD votes to class two, the final decision will be class two and so on.

### Classification performance using the SURF features

The classification system proposed can be broadly divided into five experiments, where each experiment is the input fed to the classifier. The first three experiments depend on using the SURF features of the MD, FA and RD maps individually; the fourth experiment depends on using the fusion of the SURF features of the aforementioned maps, and the fifth one depends on using the fusion of the classifier decisions of each of the aforementioned maps.

**Fig. 5:**
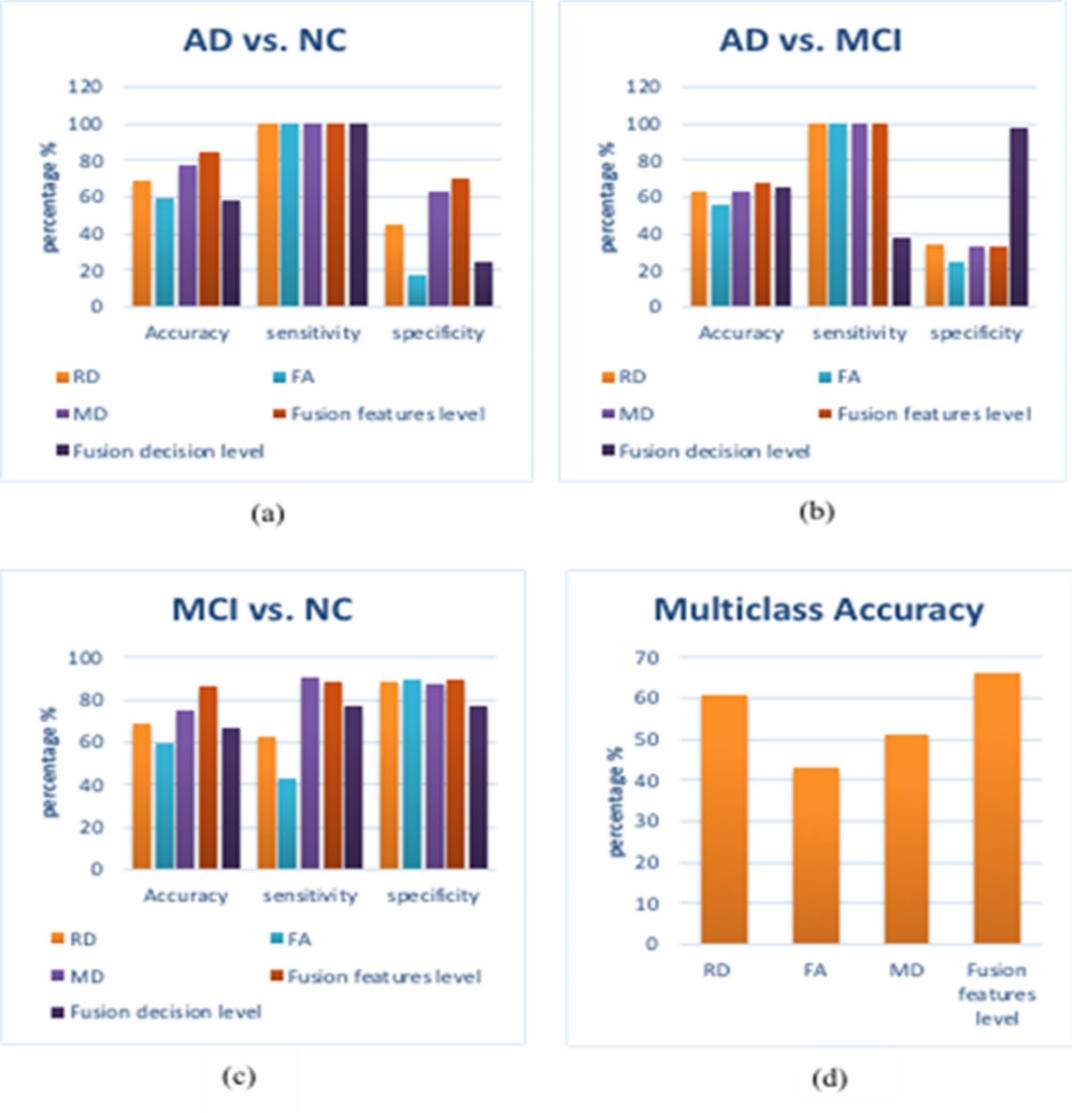
Comparison performance of SURF features: (**a**) AD vs. NC, (**b**) AD vs. MCI, (**c**) MCI vs. NC, and (**d**) Multiclass system.

Table 3; Fig. 5 show the performance results for both multi and binary classification systems. The binary classification is performed for AD vs. MCI, AD vs. NC, and MCI vs. NC. Both classification systems are done for each of the five experiments explained above. Comparing the first three experiments, the best accuracy, specificity, and sensitivity results are shown in bold while the best performance results among the other two experiments are shown in red. The performance measurements are defined within a range using the standard deviation (STD).

For using the individual maps, the RD map outperforms using FA either MD maps in multiclass and AD vs. MCI classification. It gives an accuracy of 61% in multiclass, which is better than using FA and MD maps individually, which give 43% and 51.4%, respectively. For AD vs. MCI, the accuracy reaches 63% with 100% sensitivity and 34% specificity using the RD map, which is better than the results obtained using FA and MD maps. For AD vs. NC, MD map enhances the performance giving an accuracy of 77%, a sensitivity of 100%, and a specificity of 63%. Also, for MCI vs. NC, the MD map gives the best results among the three maps with an accuracy of 75%, a sensitivity of 90.5% and a specificity of 87%.

For using the fusion of the maps’ features, the fusion of the three maps enhances the system’s performance. The accuracy of multiclass is increased to be 66%, which means that fusion of the features of the maps is better in discriminating among the three classes (NC, MCI, and AD). Also, for the binary classification of AD vs. MCI, the fusion of the maps’ features increases the performance of the system giving an accuracy of 68%, a sensitivity of 100% and 34% specificity. However, MCI is an intermediate stage between AD and NC, and it is not easy to distinguish between them, the accuracy reaches 87% with high sensitivity and specificity. This obtained result is not considered bad for the first time using these three maps together.

For using the fusion of the maps’ decision, the fusion of the classifier decisions of each of the three maps (RD, FA, and MD) improves the performance of the system. This fusion is done for binary classification. For AD vs. MCI, an accuracy, a sensitivity and a specificity of 65.7%, 38%, and 98% are obtained while for AD vs.NC, an accuracy of 58.8%, a sensitivity of 100% and specificity of 25% are obtained. Finally, for MCI vs. NC, an accuracy of 66.7%, a sensitivity of 76.7% and a specificity of 77% are achieved.

In summary, the feature fusion gives the highest accuracy for both multi- and binary- classification. In the case of using the individual maps, the RD map achieves the best performance for the multiclass classification while the MD map achieves the best for the binary classification.

**Table 3:** Accuracy ± STD (%), sensitivity ± std (%), and specificity ± std (%) for classification system using bow based on SURF features.

SURF	Multiclass	AD vs. MCI			AD vs. NC			MCI vs. NC		
	Accuracy	Accuracy	Sensitivity	Specificity	Accuracy	Sensitivity	Specificity	Accuracy	Sensitivity	Specificity
RD	61 ± 26	63 ± 27	100	34 ± 0.3	69 ± 30.5	100	45 ± 0.4	69 ± 23	62.5 ± 0.38	88.5 ± 0.25
FA	43 ± 25.7	56 ± 25	100	25 ± 0.4	59 ± 22.6	100	17 ± 0.4	59 ± 20	43.3 ± 0.38	89.5 ± 0.25
MD	51.4 ± 18	63 ± 26.3	100	33 ± 0.4	77 ± 28	100	63 ± 0.3	75 ± 24	90.5 ± 0.17	87 ± 0.14
Feature fusion	66.2 ± 14.5	67.8 ± 16	100	33 ± 0.2	84.4 ± 12	100	70 ± 0.2	86.9 ± 13	88 ± 0.2	90 ± 0.1
Decision fusion		65.7 ± 19.9	38.3 ± 30	98 ± 6.2	58.8 ± 27	100	25 ± 40	66.7 ± 32	76.7 ± 31	77 ± 41

### Classification performance using the SIFT features

**Fig. 6:**
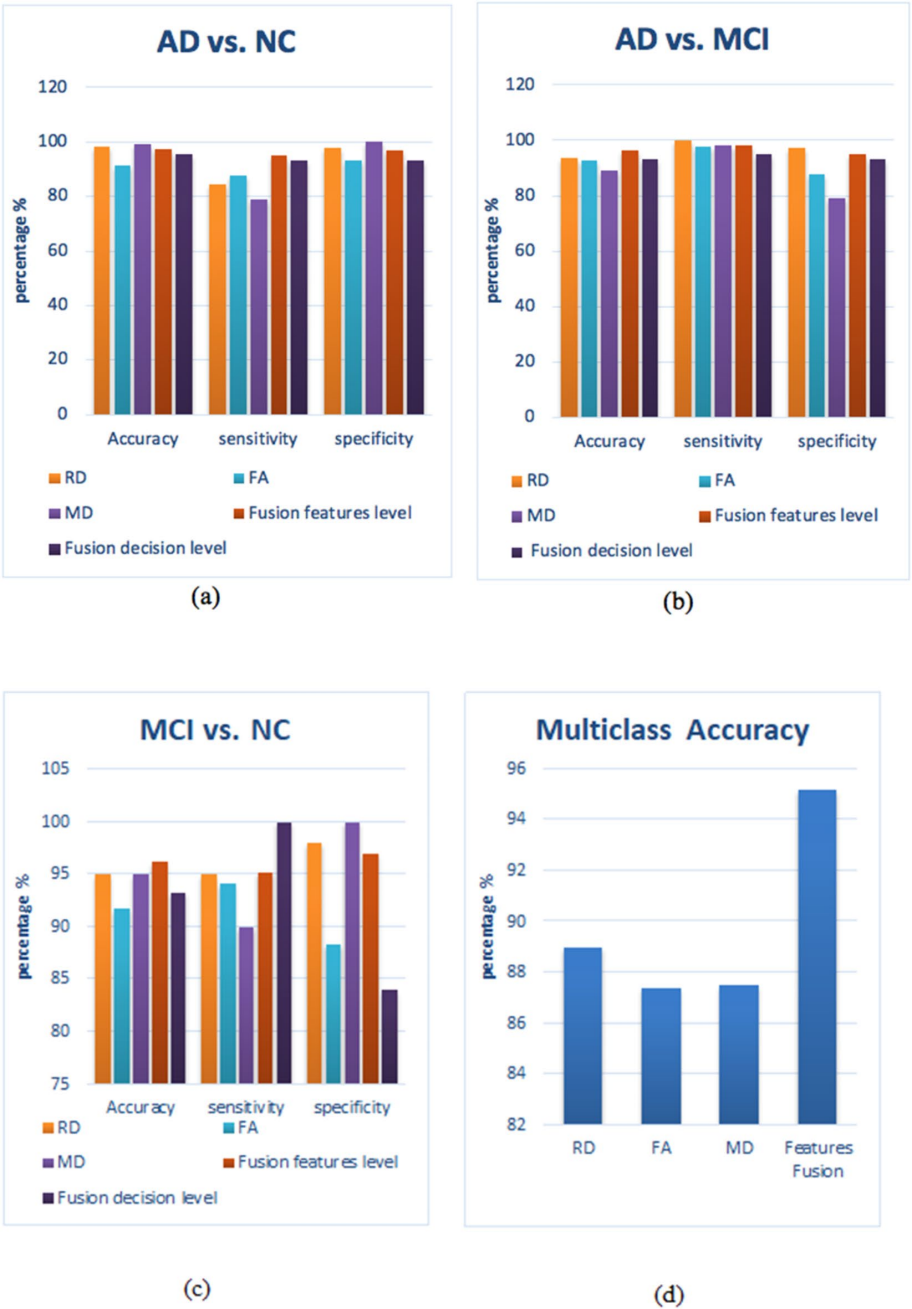
Comparison performance of SIFT features: (**a**) AD vs. NC, (**b**) AD vs. MCI, (**c**) MCI vs. NC, and (**d**) multiclass system.

The classification system using the SIFT features is also divided into five experiments, as explained in the previous section. Table 4; Fig. 6 present the accuracy, sensitivity, and specificity for multi and binary classification AD vs. MCI, AD vs. NC and MCI vs. NC based on SIFT features extraction as an entry for BoW step.

Using the RD map individually, it gives an accuracy of 89% in multiclass, which is better than accuracies obtained in case of FA and MD maps, which are 87.4% and 87.5%, respectively. For AD vs. MCI, the accuracy reaches 93.7% with 100% sensitivity and 97.1% specificity. For AD vs. NC, the RD map gives the best results among the three maps with an accuracy of 98.5%, a sensitivity of 100%, and a specificity of 98%. Finally, for MCI vs. NC, the RD map gives an accuracy of 95%, a sensitivity of 95% and a specificity of 98%. The RD map outperforms using FA either MD maps in multiclass and binary classification.

Results from the fusion of the maps’ features showed that the feature fusion enhances the performance of the classification system. The accuracy of multiclass is increased to be 95.2%. Also, for the binary classification of AD vs. MCI, the fusion of the maps’ features increases the performance of the system giving an accuracy of 96.4%, a sensitivity of 98.3%, and a specificity of 95%. As said before, MCI is not easy to distinguish it with NC, features’ fusion can differentiate between them with high accuracy of 96.2%, a sensitivity of 95.2% and specificity of 97%.

**Table 4:** Accuracy ± STD (%), sensitivity ± std (%), and specificity ± STD(%) for classification system using bow based on SIFT features.

SIFT	Multiclass	AD vs. MCI			AD vs. NC			MCI vs. NC		
	Accuracy	Accuracy	Sensitivity	Specificity	Accuracy	Sensitivity	Specificity	Accuracy	Sensitivity	Specificity
RD	89 ± 9	93.7 ± 10.4	100	97.1 ± 0.1	98.5 ± 4.5	100	98 ± 0.05	95 ± 11	95 ± 0.14	98 ± 0.08
FA	87.4 ± 6.6	92.6 ± 10.3	97.5 ± 0.079	87.67 ± 0.2	91.19 ± 10.17	87.67 ± 0.2	93 ± 0.1	91.67 ± 14.16	94.17 ± 0.12	88.33 ± 0.19
MD	87.5 ± 11	89 ± 10.9	98 ± 0.063	79 ± 0.2	99 ± 4.52	79 ± 0.2	100	95 ± 8	90 ± 0.17	100
Feature fusion	95.2 ± 3	96.4 ± 4	98.3 ± 0.03	95 ± 0.07	97.5 ± 5	95 ± 0.07	96.7 ± 0.08	96.2 ± 3.6	95.2 ± 0.06	97 ± 0.07
Decision fusion		93.3 ± 14	95 ± 15	93 ± 21	95.7 ± 27	93 ± 21	93 ± 12	93.3 ± 8	100	84 ± 22

Comparing the results together, it is shown that the fusion at features level is a good point giving relative high results in accuracy, sensitivity, and specificity which means that this fusion has a high ability to capture the leanness effectively rather than other ways as gathering features of three maps together collecting all intensities giving variety and high capability to capture the changes.

Time of execution and processing of the classification algorithm is one of the parameters that must be taken into consideration for complexity and especially if clinicians need a quick response, as illustrated in Table 5. The operating system used here in calculating the time of execution is 64-bit operating system, x64-based processor and the processor specifications are Intel^®^ Core(TM) i5-7200U CPU @2.5 GHz 2.7 GHz with 8.0 GB RAM.

**Table 5:** Time of execution of classification in seconds.

	MD map	FA map	RD map	Feature fusion	Decision fusion
Time of execution (s)	155.9	129.7	112.5	321.7	483.7

According to the reason of increasing time of fusion using the decision as in this step, it is needed to calculate the features of three maps firstly individually and then using their comparing decisions to get final one which takes more time than concatenating the features. There is another evaluation criterion to measure the performance of overall systems, which is robustness. Robustness of the system means how the system is stable and not affected by any noise or error to be during the time of execution. This Robustness of the system can be described by the AUC.

**Fig. 7:**
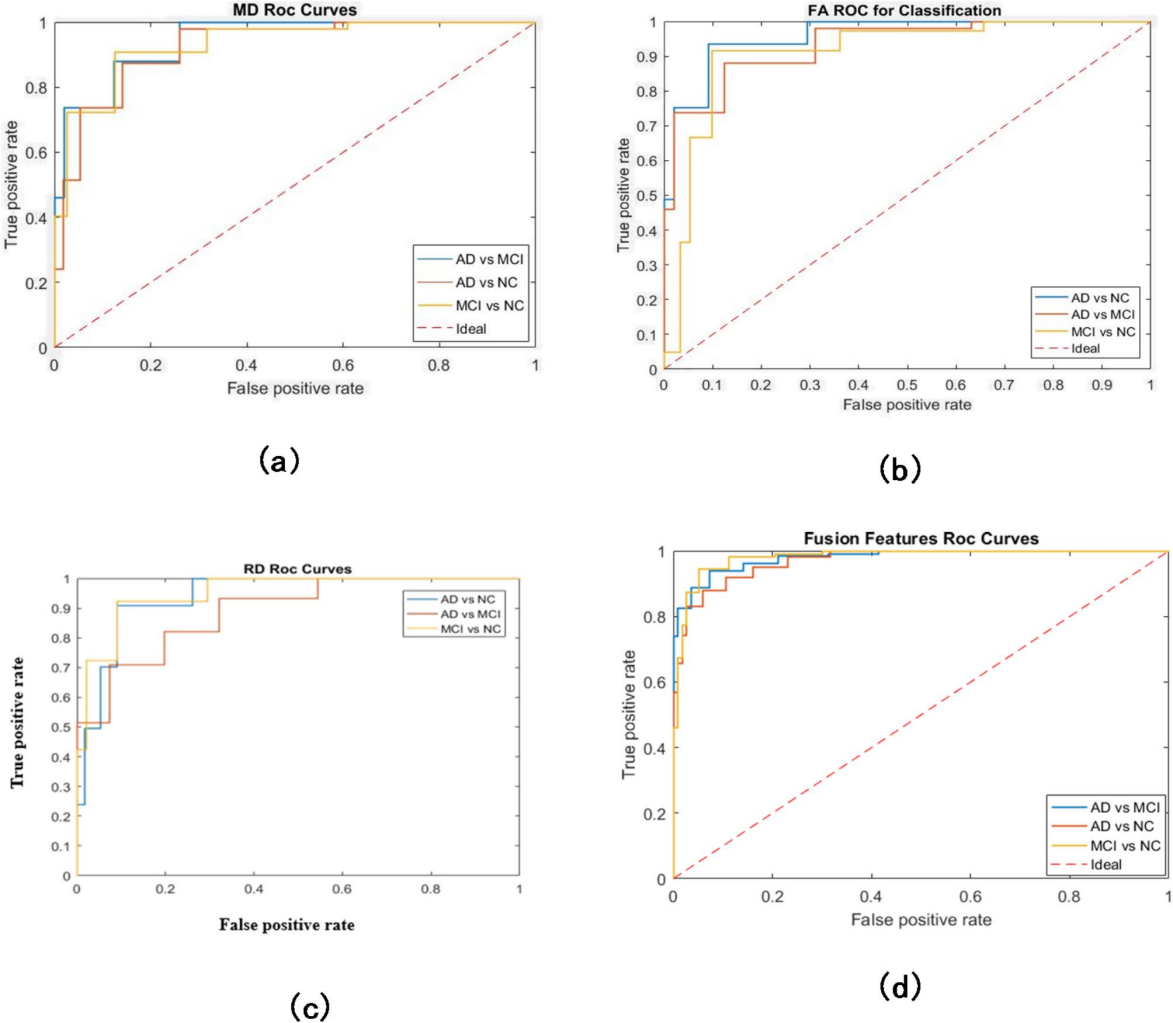
ROC curves of the maps (**a**) MD ROC curves, (**b**) FA ROC curves, (**c**) RD ROC curves, and (**d**) Features Fusion.

**Fig. 8:**
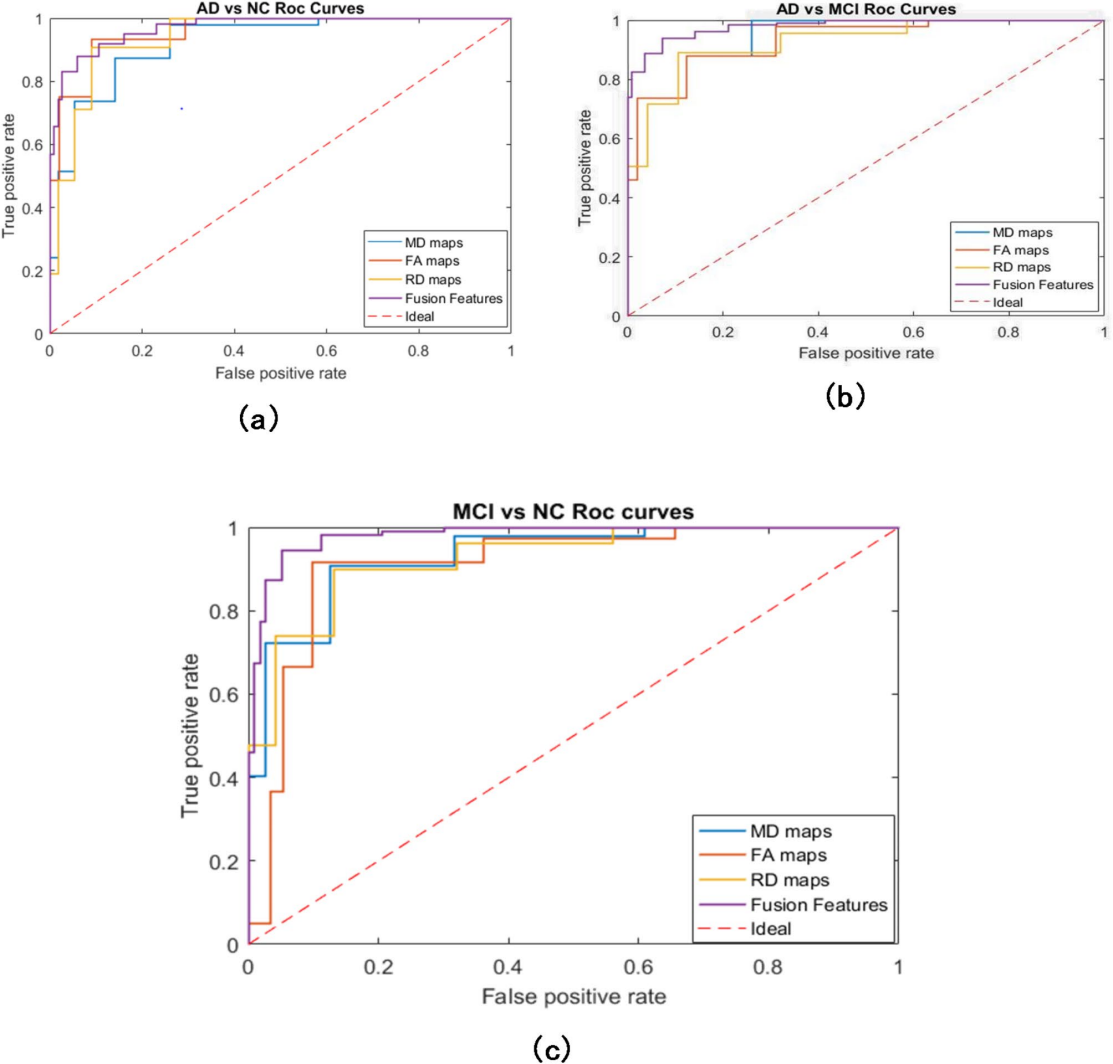
ROC curves for binary classification: (**a**) AD vs. NC, (**b**) AD vs. MCI and (**c**) MCI vs. NC.

As shown in Fig. 7, FA and RD maps are more robust than MD map if it is compared between them as a single map for extracting the features and BoW algorithm while fusing their features together enhanced the robustness of the system which ensures the results before which state that using fusion of the features level is better in accuracy, sensitivity, and specificity if it is not essential to take the time in consideration.

Also, Fig. 8 shows that fusion of the features is the best relative to the results and the most robust one obtained in this research for classifying the AD from NC and MCI with the high AUC almost 0.98.

From the previous results obtained, it is concluded that SURF features are not good and not precise enough. Since it can’t capture the small details in images to be used in the classification system. Also, fusion as a decision level in classification gives relatively good results to some point if it compared to the maps individually, and this helps to support the decision.

To our knowledge, none of the researchers before used RD nor FA maps as visual intensities values and a fusion of three maps together as features level or decision level. If it is taken into consideration the complexity and consuming time as illustrated in Table 5, this paper suggests the use of RD map individually is better and gives high performance rather than using MD and FA maps as single maps which often use in research. This is interesting and promising research.

Comparing the research results to the previous work existing in the literature, RD map and fusion of features can effectively capture the atrophy, as shown in Table 6.

**Table 6:** Proposed algorithm in comparison with the previous.

Previous works	Maps used	Features extraction method	Classifier	Accuracy
Dyrba et al.^27^	FA and MD indices	Using the plant’s approach	SVM	80% FA 83% MD
Ahmed et al.^29^	The visual appearance of MD maps	CHF	SVM	86.7% AD vs. NC 73% AD vs. MCI
Ahmed et al.^31^	MD maps	A multimodal approach using LG-CHF	SVM	90.2% AD vs. NC 77% AD vs. MCI
The proposed CAD method	The visual appearance of MD, FA, and RD maps Also Fusion of features of the three maps	BoW based on SIFT and SURF features	SVM	87.5% MD & FA multiclass 92.5% & 89% FA and MD AD vs. MCI 99 & 91% MD and FA AD vs. NC 97.5% Fusion features AD vs. NC, 89% RD multiclass, 98.5% RD AD vs. MCI

Although the interesting results our research presents. We acknowledge the limitations of our study in terms of controlling for all potential confounding variables and acknowledge that a more in-depth analysis of potential confounding variables is crucial for a comprehensive understanding of our findings. These confounding variables include: the potential impact of each of these factors on the accuracy and reliability of our findings such as **“Age**,**” “Gender**,**” “Level of education**,**” “Medication use**,**” “Comorbid conditions”.** To address this, in our study, the influence of these confounding variables, we used a strategy of age, gender, level of education **matching between groups** to mitigate the influence of these confounding variables. Moreover, **suggesting other two strategies to be considered in future research as example “Controlling for gender in the statistical analysis**,**” “Adjusting for relevant covariates in the regression models”**.

## Conclusion

This paper presents a promising classification system for Alzheimer’s disease (AD) diagnosis based on the visual signatures of hippocampus diffusion tensor imaging (DTI) maps. It extracts features from MD, FA, RD maps using powerful descriptors like SIFT and SURF, represented through the Bag of Words (BoW) model. This signature effectively captured the differences between healthy controls (NC), mild cognitive impairment (MCI), and AD patients by employing SVM for the classification task.Our key Findings: (1) The proposed system achieved impressive accuracy: based on FA maps SIFT visual signature, 89% for distinguishing MCI, AD, and NC, and 91% for AD vs. NC. (2) This multi-class classification approach goes beyond simple binary comparisons. (3) Fusion Boosts Performance: We demonstrate that feature-level and decision-level fusion of all DTI maps further improves accuracy, attaining 95% for multi-class classification and 96% for MCI vs. NC using SIFT-BoW. (4) RD Maps Emerge as New Biomarkers: Notably, RD maps, rarely used in other studies, show promising results with 86% accuracy individually and contribute significantly to improved accuracy when fused with other maps. While SIFT and SURF are powerful, they are computationally intensive and may not capture the full complexity of Alzheimer’s-related changes compared to deep learning methods. They are often used in combination with other techniques to improve accuracy and robustness. In summary, SIFT and SURF features are important in Alzheimer’s diagnosis because they provide robust, invariant, and interpretable features that can capture subtle structural changes in the brain. They are particularly useful when combined with other machine learning or deep learning approaches to enhance classification performance. The manuscript suggests some future directions such as: (1) Investigate the impact of gender on signature characteristics. (2) investigate the impact of scanner variability on the findings. This could involve analyzing data from multiple scanners or employing scanner-specific correction methods. (3) Explore the fusion of different anisotropy maps to improve classification accuracy. (4) Analyze other potential regions of interest (ROIs) like the amygdala. (5) Add comparative experiments with other advanced methods, such as RF, XGBoost, deep learning methods, other feature extraction and fusion methods, to comprehensively evaluate the superiority and shortcomings of the proposed method. (6) Deep learning potential: Employing deep learning for feature extraction holds promise for further performance improvement. 5) Multi-modal fusion: Combining DTI with other MRI modalities like sMRI and fMRI could unlock even greater accuracy and insights.

This proposed system paves the way for improved AD diagnosis by utilizing not only established biomarkers like MD but also previously underutilized RD maps. The promising results and suggested future directions point towards a future where accurate and early diagnosis of AD becomes a reality, ultimately leading to better patient care and treatment strategies.

## Electronic supplementary material

Below is the link to the electronic supplementary material.

Supplementary Material 1

## Data Availability

The dataset used in this study was obtained from ADNI Website (Public available data). The data that support the findings of this study are available on reasonable request from the ADNI (http://adni.loni.usc.edu/).
