# Supplementary material for: Classification method based on surf and sift features for alzheimer diagnosis using diffusion tensor magnetic resonance imaging
Source: Sci Rep. 2025 Mar 21;15:9782. doi: 10.1038/s41598-025-92759-2 (PMC11928662; doi:10.1038/s41598-025-92759-2)
Supplement: Supplementary file 1 — Supplementary Material 1 [file 41598_2025_92759_MOESM1_ESM.docx]

**Nomenclature**

| **AD  ADNI**  **AUC**  **BoW  CAD**  **CBIR**  **CDSS**  **CHF**  **CT CSF**  **DoF DoG  DTI**  **DW FA**  **FDA**  **FN**  **FoV FP**  **fMRI**  **FSL  GM**  **Libsvm  MCI**  **MD**  **md-aMCI**  **MDPs  MMSE  MRI  NC**  **NIBIB**  **NMR PET**  **pMCI**  **RBF**  **RF**  **ROC**  **ROI**  **sd-aMCI**  **sd-fMCI**  **SE**  **SIFT**  **sMCI**  **sMRI  SPECT**  **SPM**  **STD SURF  SVM**  **TBSS**  **TE**  **TI**  **TN**  **TP**  **TR**  **VBA**  **VBM**  **WM** | Alzheimer’s Disease.  AD Neuroimaging Initiative.  Area Under the Curve.  Bag-of-Words.  Computer-Aided Diagnosis.  Content-Based Image Retrieval.  Clinical Decision Support System.  Circular Harmonic Function  Computed Tomography.  Cerebrospinal Fluid.  Degree of Freedom. Difference of Gaussian.  Diffusion Tensor Imaging.  Diffusion-Weighted.  Fractional Anisotropy.  Food and Drug Administration  False Negatives.  Field of View.  False Positives.  Functional MRI.  FMRIB Software Library. Gray Matter.  Library of Support Vector Machine.  Mild Cognitive Impairment.  Mean Diffusivity.  Multiple Domains MCI.  Maximum Density Paths.  The Mini-Mental State Examination.  Magnetic Resonance Imaging.  Normal Control.  National Institute of Biomedical Imaging and Bioengineering.  Nuclear Magnetic Resonance.  Positron Emission Tomography.  Progressive MCI.  Radial Basis Function.  Radiofrequency.  Receiver Operating Curve.  Region of Interest.  Single Domain Amnestic MCI.  Single Domain Frontal MCI.  Spin Echo Sequence.  Scale Invariant Feature Transform.  Stable MCI.  Structural Magnetic Resonance Imaging.  Single-Photon Emission Computerized Tomography.  Statistical Parametric Mapping.  Standard Deviation.  Speed Up Robust Feature.  Support Vector Machines.  Tract-Based Spatial Statistics.  Echo Time.  Inversion Time.  True Negatives.  True Positives.  Repetition Time.  Voxel-Based Analysis.  Voxel-Based Morphometry.  White Matter. |
| --- | --- |
